# The Antimicrobial and Immunomodulatory Function of RNase 7 in Skin

**DOI:** 10.3389/fimmu.2019.02553

**Published:** 2019-11-05

**Authors:** Franziska Rademacher, Sylvia Dreyer, Verena Kopfnagel, Regine Gläser, Thomas Werfel, Jürgen Harder

**Affiliations:** ^1^Department of Dermatology, University of Kiel, Kiel, Germany; ^2^Division of Immunodermatology and Allergy Research, Department of Dermatology and Allergy, Hannover Medical School, Hanover, Germany; ^3^Hannover Unified Biobank, Hannover Medical School, Hanover, Germany; ^4^Cluster of Excellence RESIST (EXC 2155), Hannover Medical School, Hanover, Germany

**Keywords:** RNase 7, antimicrobial ribonucleases, RNA, innate immunity, cutaneous defense, skin infection, skin inflammation

## Abstract

The human ribonuclease RNase 7 has been originally isolated from human skin and is a member of the human RNase A superfamily. RNase 7 is constantly released by keratinocytes and accumulates on the skin surface. The expression of RNase 7 in keratinocytes can be induced by diverse stimuli such as cytokines, growth factors, and microbial factors. RNase 7 exhibits a potent broad spectrum of antimicrobial activity against various microorganisms and contributes to control bacterial growth on the skin surface. The ribonuclease and antimicrobial activity of RNase 7 can be blocked by the endogenous ribonuclease inhibitor. There is also increasing evidence that RNase 7 exerts immunomodulatory activities and may participate in antiviral defense. In this review, we discuss how these characteristics of RNase 7 contribute to innate cutaneous defense and highlight its role in skin infection and inflammation. We also speculate how a potential dysregulation of RNase 7 promotes inflammatory skin diseases and if RNase 7 may have therapeutic potential.

## Introduction

Human skin is permanently in contact with microorganisms. Among these microorganisms are many beneficial members of the microbiota. In addition, the skin has to deal with potential pathogenic microorganisms causing partially severe skin infections. There is increasing evidence that antimicrobial peptides and proteins (AMP) control the growth of microorganisms on all epithelial tissues including skin. AMP may play an important role to shape and control the preferred microbiota as well as to protect the skin from pathogenic microorganisms. Human skin is able to produce different types of AMP that exhibit individual activity profiles. One major AMP expressed by keratinocytes and released on the skin surface is the ribonuclease RNase 7. RNase 7 belongs to the human RNase A superfamily which consists of 13 genes located on chromosome 14 and contains eight different members with RNase activities (RNase 1–8) (1). The RNase A superfamily is named after its founding member, bovine pancreatic ribonuclease (RNase A). The members of this family contain an RNase catalytic domain that is composed of one lysine and two histidine residues. The protein structure is characterized by eight cysteine residues which are connected via four disulfide bridges (1). A huge repertoire of diverse physiological functions of these RNases has been reported during the last decades. Examples are angiogenic, neurotoxic, and diverse immunomodulatory activities (2). Several members of the human RNase A superfamily are also characterized by antimicrobial activities and there is increasing evidence that they participate in innate immunity. In particular, RNase 2 (also termed eosinophil-derived neurotoxin, EDN) and RNase 3 (also termed eosinophil cationic protein, ECP) exhibit diverse antiviral and antibacterial activities as well as immunomodulatory activities suggesting an important role in innate host defense (3, 4). Moreover, antimicrobial activities have also been reported for RNase 5 (also named angiogenin due to its angiogenic potency) (4–6), RNase 6 (7), RNase 7 (8, 9), and RNase 8 (10). RNase 5 and RNase 7 are both expressed by keratinocytes and thus may contribute to cutaneous innate defense (6, 11). Indeed, there is increasing evidence that RNase 7 may play an important role to protect skin from infection (8). This review aims to summarize the current knowledge about the physiological activities of RNase 7 and to highlight its role in cutaneous host defense.

## Expression and Induction of RNase 7 in Skin

### Site of RNase 7 Expression

The RNase 7 protein has been originally isolated from stratum corneum extracts during an attempt to identify and characterize the antimicrobial factors produced by human healthy skin (12). As the name implies, it is the seventh member of the human RNase A superfamily and exhibits ribonuclease activity. It has a molecular mass of 14.5 kDa and is highly cationic due to the presence of many arginine and lysine residues (12). Isolation of the protein from human skin and subsequent cloning of the corresponding cDNA enabled the identification of RNase 7 gene expression in human skin-derived keratinocytes (12). Of note, RNase 7 is not only expressed in human skin. Gene expression analyses revealed expression in various tissues such as heart, liver, kidney, pharynx, and tonsil (12, 13). In addition, the group of Spencer et al. identified a major role of RNase 7 in kidney and bladder host defense (14, 15).

Immunostaining of skin biopsies revealed RNase 7 expression in the keratinocytes throughout all epidermal layers with an increased staining in the more differentiated uppermost layers and an accumulation in the stratum corneum (12, 16). These data are in concordance with expression analyses of cultured keratinocytes demonstrating a higher RNase 7 gene expression in differentiated cultures as compared with proliferating keratinocytes (6). RNase 7 immunoreactivity was also present in sebaceous glands and hair follicles (12, 17). Analysis of skin rinsing fluids revealed the presence of RNase 7 throughout various body areas confirming the constitutive expression and release of RNase 7 by keratinocytes (16, 18). Interestingly, RNase 7 is also expressed in fetal skin suggesting that RNase 7 may already control cutaneous microbial growth of the fetus and newborn (19). In addition, RNase 7 may have other, as yet unknown functions in fetal skin such as a regulatory role in development. However, this hypothesis remains to be proven.

### Induction of RNase 7 Expression

**Figure 1:**
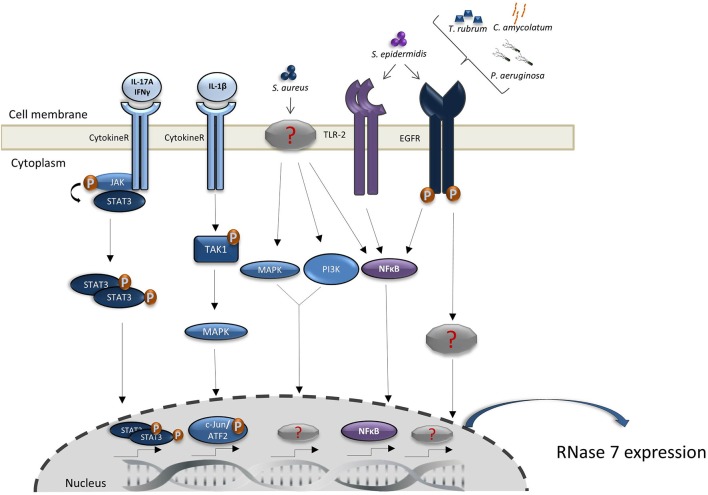
Regulation of RNase 7 induction. Shown are the proposed signal transduction pathways implicated in the induction of RNase 7 by microorganisms and cytokines. Detailed explanations are given in the main text.

In addition to the constitutive expression, RNase 7 expression in keratinocytes can be induced by various stimuli and different signal transduction pathways (summarized in Figure 1). Proinflammatory cytokines such as IL-17A, interferon-gamma and IL-1 are able to induce the expression of RNase 7 in keratinocytes (12, 17, 20, 21). Especially the synergistic action of proinflammatory cytokines such as IL-17A and interferon-gamma markedly induced the expression of RNase 7 (20). The synergistic induction of RNase 7 by IL-17A/interferon-gamma was mediated by the signal transducer and activator of transcription 3 (STAT3) (20). Mohammed et al. reported that the induction of RNase 7 by IL-1beta in corneal epithelial cells was mediated by TGFbeta-activated kinase-1 (TAK-1). This in turn led to activation of the mitogen-activated protein kinase (MAPK) pathway resulting in activation of the transcription factors c-Jun and activating transcription factor 2 (ATF2). In contrast, the NF-kappaB pathway had no major influence on the IL-1beta-mediated induction of RNase 7 in corneal epithelial cells (21). Induction of RNase 7 by pro-inflammatory cytokines was also detected in the keratinocytes cell line HaCaT (22). Overall, the induction of RNase 7 by proinflammatory cytokines suggests that the local environment of inflammatory skin diseases like psoriasis trigger the increased expression of RNase 7 (23, 24).

Since RNase 7 exhibits potent antimicrobial activity (see below), it would be of biological significance that keratinocytes respond with an increased RNase 7 expression to the presence of microorganisms, in particular potential pathogens. Indeed, the expression of RNase 7 in keratinocytes can be induced by *Staphylococcus (S.) aureus* (25–28), *Pseudomonas (P.) aeruginosa* (29), *Enterococcus (E.) faecium* (16), and the dermatophyte *Trichophyton (T.) rubrum* (30). Of note, also skin commensals such as *Staphylococcus (S.) epidermidis* and *Corynebacterium (C.) amycolatum* induce RNase 7 expression in keratinocytes indicating that the presence of commensals leads to an increased RNase 7 production thereby strengthening cutaneous defense (22, 27, 31). *S. epidermidis* also enhanced the *S. aureus*-induced expression of RNase 7 in keratinocytes suggesting that the presence of commensals amplifies the defense response of human keratinocytes toward pathogens such as *S. aureus* (27). Interestingly, the induction of RNase 7 by the skin commensals *S. epidermidis* (27) and *C. amycolatum* (31), and by *P. aeruginosa* (29) and *T. rubrum* (30) depends on the involvement of the epidermal growth factor receptor (EGFR). This suggests that the EGFR plays an important role in cutaneous defense by its crucial role to mediate the expression of AMP such as RNase 7 and may offer an explanation for the increased susceptibility for skin infections of cancer patients receiving anti-EGFR therapy (32). Wanke et al. reported that—in addition to the EGFR—also Toll-like receptor-2 (TLR-2) and the transcription factor NF-kappaB are involved in the induction of RNase 7 in keratinocytes simulated with culture supernatants of *S. epidermidis*. This is in contrast to *S. aureus* which has been reported to activate the MAPK and phosphatidylinositol 3-kinase/AKT signaling pathways to induce RNase 7 expression (27). Thus, it seems that commensal and pathogenic bacteria activate different signal transduction pathways to induce RNase 7 expression in keratinocytes. RNase 7 expression in keratinocytes can also be induced by *Borrelia burgdorferi*, a bacterium that causes Lyme borreliosis and is transmitted by *Ixodes* ticks. Interestingly, tick saliva proteins have been shown to inhibit the *Borrelia burgdorferi-*mediated induction of RNase 7 and of other antimicrobial peptides and chemokines. This suggests that components of the tick saliva inhibit cutaneous innate defense reactions against *Borrelia burgdorferi* and thereby help the bacterium to evade local host defense and to disseminate into the body (33).

The role of cutaneous RNase 7 expression in the context of viral infection is less explored. It has been reported that keratinocytes infected with dengue virus showed an increased RNase 7 expression. If this is a direct induction or indirectly mediated by the release of inflammatory mediators is not clear. In addition, if RNase 7 influences the infectivity by dengue virus has not been reported (34). UV-B radiation also induces the expression of RNase 7 and other AMP in keratinocytes. This induction may contribute to the UV-mediated strengthening of the innate immune response (35). Niacinamide has also been identified as an inducer of the expression of RNase 7 and other antimicrobial peptides in human keratinocytes. Niacinamide is a well-known cosmetic ingredient and its antimicrobial peptides-inducing capacity may account for its observed beneficial effects on the skin barrier (36).

## Antimicrobial and Ribonuclease Activity

### Antimicrobial Spectrum of RNase 7

A key feature of RNase 7 is its high antimicrobial activity against a wide spectrum of microorganisms. *In vitro* studies showed that RNase 7 is highly antimicrobial effective in low micromolar concentrations against Gram-positive and Gram-negative bacteria like *S. aureus, P. aeruginosa, C. amycolatum, E. faecium, Mycobacterium vaccae*, the yeast *Candida (C.) albicans* and *Pichia pastoris* and the dermatophyte *T. rubrum* (12, 16, 30, 37–41). The known microorganisms susceptible to RNase 7 are listed in Table 1 (6, 7, 12, 14–16, 25, 29–31, 37–50). The functional relevance of the antimicrobial activity of RNase 7 and its contribution to the antimicrobial capacity of stratum corneum was demonstrated by the use of antibodies that neutralized the antimicrobial activity of RNase 7. Using such antibodies, we could show that inactivation of RNase 7 in human stratum corneum extracts led to an increased outgrowth of *P. aeruginosa, S. aureus, C. amycolatum*, and *E. faecium*. These results demonstrated that RNase 7 is part of the growth control mechanism in the stratum corneum (16, 25, 29, 31).

**Table 1:** Overview of the microorganism susceptible to RNase 7.

**Microorganism**	**References**
**GRAM-POSITIVE BACTERIA**	
*Staphylococcus aureus*	(12, 25, 37, 38, 42–46)
*Staphylococcus saprophyticus*	(7, 47)
*Enterococcus faecium*	(6, 12, 16, 44)
*Enterococcus faecalis*	(6, 7, 48)
*Propionibacterium acnes*	(12)
*Micrococcus luteus*	(44)
*Corynebacterium amycolatum*	(31)
*Corynebacterium xerosis*	(31)
**GRAM-NEGATIVE BACTERIA**	
*Pseudomonas aeruginosa*	(12, 14, 29, 37, 44, 48, 49)
*Escherichia coli*	(7, 12, 14, 15, 37, 44, 46–48)
*Proteus mirabilis*	(13, 22)
*Acinetobacter baumannii*	(44)
*Klebsiella pneumoniae*	(14)
**MYCOBACTERIA**	
*Mycobacterium tuberculosis*	(50)
*Mycobacterium vaccae*	(40)
**YEAST**	
*Candida albicans*	(12, 37, 41)
*Pichia pastoris*	(37)
**DERMATOPHYTES**	
*Trichophyton rubrum*	(30, 39)
*Trichophyton mentagrophytes*	(39)
*Microsporum canis*	(39)
*Epidermophyton floccosum*	(39)

The Gram-positive bacterium *S. aureus* is a principal skin pathogen causing many infectious cutaneous diseases. Our work with *ex vivo* skin explants infected with *S. aureus* showed an increased release of RNase 7 expression. Inhibition of the antimicrobial activity of RNase 7 by specific antibodies resulted in considerable outgrowth of *S. aureus* on the skin surface. These results accentuate the functional relevance of RNase 7 in cutaneous defense against hazardous skin pathogens (25). This goes in line with a study from Zanger et al. who analyzed the RNase 7 expression levels of unaffected skin from healthy control persons and subjects with a *S. aureus* skin infection after a journey to a tropic or subtropic destination. They detected a 64% higher RNase 7 expression level in skin of the control group than in the unaffected skin of the infected persons. These data suggest that high RNase 7 baseline levels in healthy skin provide an increased protection against *S. aureus* infection (51).

### Antimicrobial Mechanisms of RNase 7 and Influence of the Ribonuclease Inhibitor

As described above, RNase 7 is a member of the RNase A superfamily and a potent ribonuclease that is able to degrade RNA. This raises the question of whether the ribonuclease activity is essential for the antimicrobial activity of RNase 7. Huang et al. designed RNase 7 mutants (H15A, K38A, and H123A) lacking ribonuclease activity and showed that these mutants are still able to kill *P. aeruginosa*. In addition, they identified three clusters of cationic residues on the surface of RNase 7. Substitution experiments of cationic residues identified the first N-terminal cluster with the lysine residues K1, K3, K111, and K112 as essential for antimicrobial activity. The other two clusters located on rigid secondary structures did not influence antimicrobial activity of RNase 7 (37). In general, the N-terminal cluster of RNases from the RNase A superfamily is the conserved region responsible for their antimicrobial activity (44). In line with the ribonuclease-independent killing activity against *P. aeruginosa*, we constructed a recombinant RNase 7 mutant without ribonuclease activity and showed that this mutant was still able to kill *E. faecium* (16). Ribonuclease-independent antibacterial activity has also been described for RNase 3/ECP (52).

The ribonuclease activity of RNases from the RNase A superfamily can be inhibited by binding to the 50 kDa, cytosolic, horseshoe-shaped Ribonuclease Inhibitor (RI) in a 1:1 molar ratio. The RI forms tight complexes with the RNases which is known as one of the tightest formation in biological systems (53). It is an obvious question if binding of RI influences the antimicrobial activity of RNase 7. Addition of RI to RNase 7 suspensions and subsequent testing of the killing capacity of RNase 7 toward *C. albicans* and *E. faecium* resulted in a reduced antimicrobial activity indicating that the RI blocks the antimicrobial activity of RNase 7 (6). This inhibition may be due to steric interaction and not due to specific inhibition of the ribonuclease activity because the ribonuclease activity is not essential for antibacterial killing as described above. In concordance with these results, Spencer et al. also reported that the RI bound to RNase 7 and inhibited its antimicrobial activity by blocking its ability to bind the cell wall of uropathogenic bacteria (54).

Immunohistochemical analysis revealed that the RI is located mainly in the suprabasal epidermal layers but is absent in the stratum corneum. In addition, incubation of the RI with stratum corneum extracts led to the degradation of the RI suggesting that proteolytic activity of the stratum corneum degrades the RI (6). In contrast to the RI, RNase 7 is abundant in the stratum corneum. This led to the hypothesis that in the epidermis RNase 7 is complexed with the RI and thereby inactivated. Degradation of the RI in the stratum corneum hinders RI-mediated inactivation of RNase 7 thereby liberating RNase 7 to function as antimicrobial factor (6, 55).

The exact mechanisms underlying the antimicrobial activity of RNase 7 are not fully understood. Huang and coworkers performed binding experiments with DNA-binding SYTOX^®^ Green dye and showed that RNase 7 is able to bind to the negatively charged membrane of *P. aeruginosa* and permeabilize the bacterial membrane (37). Torrent et al. demonstrated by means of liposome leakage assays that RNase 7 provokes membrane disruption by binding to bacterial membranes. Further experiments showed that RNase 7 did not interact with uncharged liposomes. Because of this fact the authors deduced that this membrane interaction is electrostatically driven (56). Further bacterial studies by this group showed a high leakage affinity of RNase 7 for *S. aureus* and *E. coli* membranes. RNase 7 depolarized the membrane by binding to lipopolysaccharide (LPS) and peptidoglycan, both major components of bacterial cell membranes from Gram-positive and Gram-negative bacteria, respectively (46). Another study from Lin et al. identified OprI (outer membrane protein I), an outer membrane lipoprotein from *P. aeruginosa*, as the initial binding site for RNase 7 instead of LPS. Addition of exogenous OprI or an anti-OprI antibody inhibited the antimicrobial activity of RNase 7 against *P. aeruginosa*. The authors conclude that upon RNase 7 binding, OprI internalizes along with RNase 7 into the cell leaving the cell membrane permeable to metabolites (49). These studies suggest that the interaction of RNase 7 with bacteria could be based on a target specific accumulation to different bacterial lipoproteins. This hypothesis needs to be proven in further studies with other bacteria.

In addition to its antibacterial activity, RNase 7 exhibits also activity against the yeast *C. albicans*. Salazar et al. described a dual mode of action for RNase 3 and RNase 7 as antifungal proteins against *C. albicans*. They designed mutants of both RNases by depleting the active catalytic site at His15 which is the complement to His12 and performed depolarization and permeabilization membrane assays to assess the antimicrobial action against *C. albicans*. They could show that both RNases act first as membrane lytic proteins followed by enzymatic cellular RNA degradation (41).

## Immunomodulatory Activities

Besides its antimicrobial activity, some studies provide evidence for an additional immunomodulatory function of RNase 7. Several members of the RNase A superfamily are well-known for their immunomodulatory activities (2). Although the role of RNase 7 as an immunomodulatory mediator is still emerging, there is first evidence of immunomodulatory functions associated with RNase 7. Resident and transiently migrating cells of the immune system are present in healthy human skin. With regard to antimicrobial defense, plasmacytoid dendritic cells (pDCs) are considered to play a key role within the types of the dermal immune system. pDCs usually circulate in the blood stream and are present in lymph nodes. Under inflammatory conditions they are able to infiltrate the skin (57). Due to their ability to secrete a several times stronger interferon-alpha (IFNα) response than any other cell types (58, 59), pDCs are crucial for cellular antimicrobial defense. A recent study from Kopfnagel et al. showed that RNase 7 in combination with human self-DNA activates a potent IFNα response in pDCs (60). This finding is in accordance with the known IFNα-inducing ability of LL-37 (61) and hBD-2/-3 (62) in complex with self-DNA. In addition, comparative experiments revealed a markedly stronger IFNα production in response to RNase 7 mixed with self-DNA as compared to stimulation with LL-37 or hBD-2 in mixture with self-DNA (60). Of note, the amount of the RNase 7-induced IFNα release was high enough to efficiently protect human keratinocytes from herpes simplex type I (*HSV-I*) infection. This was of special significance because RNase 7 itself exhibits no direct antiviral activity against *HSV-I*. In summary, this study revealed the ability of RNase 7 to convert usually not immunogenic self-DNA into a danger signal which enables a strong immunomodulatory response (60). It remains to be shown if these characteristics of RNase 7 may be linked with auto-inflammatory diseases.

T cells are present in human skin under healthy and inflammatory conditions. In healthy human skin they are of importance in wound healing, stimulate production of AMP by keratinocytes and influence keratinocyte development (63). Under inflammatory conditions T cells present in the blood can rapidly infiltrate the skin and produce pro-inflammatory cytokines, like IL-4 or interferon-gamma (64). A study of Kopfnagel et al. investigated the influence of RNase 7 on Th2 cytokine production by human CD4+T cells and Th2 cells. They reported that RNase 7 stimulation lead to a significantly reduced Th2 cytokine release of IL-4, IL-5, and IL-13. This downregulation of Th2 cytokines was found to be mediated by a reduced activation of the transcription factor GATA3. Interestingly, the ribonuclease activity of RNase 7 was dispensable for this effect. Due to this specific regulation the authors assumed a yet unknown receptor-mediated process which needs to be elucidated in further studies (65).

A novel study documents that RNase 7 mediates recognition of self-DNA by human keratinocytes. Activation of keratinocytes by a DNA/RNase 7 complex resulted in a markedly increased release of the chemokine IP-10 (CXCL10), a process that was mediated by type I interferons. In addition, the stimulation of keratinocytes with RNase 7 and DNA induced an interferon-beta (IFNß) dependent antiviral response which was sufficient to counteract an infection of the keratinocytes with herpes-simplex virus 1 (HSV-1). Taken together, there is increasing evidence that RNase 7 is able to bind self-DNA immediately after its release and induces a rapid DNA-mediated activation of keratinocytes (66) and pDCs (60). Thus, RNase 7 may serve as an alarmin which detects a disruption of the skin barrier by converting released self-DNA into a danger signal (66).

Various immunomodulatory activities of other RNases of the human RNase A superfamily have been described. Examples are processing and clearance of RNA, activation of immune cells, chemotactic activities, angiogenesis and neo-vascularization, tissue remodeling and repair, wound healing activity, and induction of apoptosis [reviewed in (67)]. Thus, it is likely that RNase 7 exhibits additional, as yet unknown, immunomodulatory functions. The known immunomodulatory activities of RNase 7 are summarized in Table 2 (60, 65, 66). Since the expression of RNase 7 is induced in inflammatory skin diseases such as psoriasis and atopic dermatitis (see below) it is likely that the immunomodulatory activities of RNase 7 may play a role in these skin diseases. Furthermore, it is also of interest to evaluate if the ribonuclease activity of RNase 7 may play a role in degrading host RNA released from damaged cells thereby controlling RNA-mediated inflammation during skin injury.

**Table 2:** Overview of the main findings regarding immunoregulatory activity of RNase 7.

**Findings**	**References**
Up-regulation of IFNα by human pDCs stimulated with RNase 7 in mixture with self-DNA	(60)
Down-regulation of Th2 cytokines (IL-4, IL-13, and IL-5) by activated human RNase 7-stimulated Th2 cells and CD4+ T cells	(65)
RNase 7 stimulation of activated T cells results in a reduced activity of the transcription factor GATA3	(65)
RNase 7 mediates sensing of self-DNA in human keratinocytes leading to an antiviral immune response	(66)

## Role of RNase 7 in Skin Diseases

### Atopic Dermatitis

It has long been assumed that in the chronic inflammatory skin disease atopic dermatitis (AD) the expression of AMP including RNase 7 is impaired. This is based on the observation that a Th2 cytokine predominance has been shown to negatively regulate AMP expression (68, 69). In addition, comparative studies showed a reduced expression of AMP in AD skin as compared to psoriasis skin (70). However, especially for RNase 7, growing evidence reveals an increased expression in AD skin as compared to healthy skin. By investigating skin biopsies using quantitative real-time PCR, Gambichler et al. showed a significantly higher expression of RNase 7 mRNA as compared to expression in healthy skin (71). In addition, Harder et al. investigated the protein expression of RNase 7 in skin biopsies using immunostaining and ELISA of skin-derived washing fluids. Both methods revealed an enhanced RNase 7 expression and secretion in lesional skin of AD patients as compared to healthy controls (23). In another study, Clausen et al. investigated RNase 7 protein expression in the uppermost skin layers using tape stripping followed by ELISA measurements. In line with the above mentioned studies they also found a higher expression of RNase 7 in AD skin as compared to healthy skin (72). Taken together, there is consistent evidence that expression of RNase 7 is induced in the skin of atopic dermatitis patients. The consequence of this induction on diseases progression remains to be analyzed in future studies.

As mentioned above, RNase 7 is able to downregulate Th2 cytokine production in CD4+ T cells. This effect was reduced in CD4+ T cells derived from AD patients (65). These data suggest that RNase 7 plays an important role to negatively regulate the expression of Th2 cytokines, a function that may be disturbed in AD thereby promoting a Th2 cytokine environment.

Filaggrin is an essential component of the skin barrier. A deficiency of this protein can lead to an impaired skin barrier function and this is assumed to be a major predisposing factor in the development of AD (73). van Drongelen et al. used 3D epidermal models with filaggrin knockdown and infected these with methicillin-resistant *S. aureus* (MRSA) bacteria. They showed that IL-31 favored epidermal *S. aureus* colonization by preventing the *S. aureus*-induced expression of RNase 7 and other AMP (26). IL-31 is a pruritus-causing cytokine with increased presence in AD (74). Thus, enhanced levels of IL-31 in AD may locally inhibit an adequate induction of RNase 7 in *S. aureus*-infected areas.

### Psoriasis

A high-performance liquid chromatography (HPLC)-based analysis detected high amounts of RNase 7 and other AMP in psoriatic scale extracts (24). In line with these data, analyses of RNase 7 protein expression by immunohistochemistry and ELISA revealed increased expression of RNase 7 in the lesional skin of psoriasis patients (23). The increased amount of RNase 7 and other AMP in psoriasis may offer an explanation as to why psoriasis patients do not often suffer from skin infections despite the disturbed skin barrier (75).

One characteristic of psoriasis is the enrichment of pDCs in lesional skin and their key role in driving the development of the disease by the release of IFNα (76). As mentioned above, Kopfnagel et al. showed that RNase 7 in complex with self-DNA is a potent trigger of pDC-derived IFNα. Thus, an enhanced production of RNase 7 may trigger inflammation in psoriasis through increased production of IFNα (60). Similarly, the ability of RNase 7 to activate an inflammatory and antiviral response in keratinocytes in the presence of self-DNA may have implications in skin diseases such as psoriasis and atopic dermatitis where self-DNA derived from injured cells may be present in increased amounts (66).

### Dermatomycoses

Major fungal cutaneous infections are superficial tinea and pityriasis versicolor which are caused by dermatophytes and *Malassezia* spp., respectively. An immunohistochemistry study from Brasch et al. investigated epidermal AMP expression in skin biopsies from infected and healthy persons. RNase 7 staining was significantly more often positive in the stratum granulosum of tinea than in the stratum granulosum of pityriasis versicolor and normal skin (77). In concordance with these findings, Firat et al. detected a high upregulation of RNase 7 expression in keratinocytes infected with *T. rubrum* (30). By blocking the EGFR, the *T. rubrum*-mediated RNase 7 induction in keratinocytes was significantly reduced. Interestingly, patients with anti-EGFR therapy have a high prevalence of cutaneous infections including those caused by dermatophytes such as *T. rubrum* (78). One may speculate that the anti-EGFR therapy may impair antifungal defense of the skin by inhibiting production of AMP such as RNase 7.

### *S. aureus* Skin Infections

As already described above, RNase 7 exhibits potent *in vitro* anti-staphylococcal activity and *ex vivo* as well as *in vivo* studies highlight an important role of RNase 7 to control the growth of *S. aureus*. This gives rise to the speculation that RNase 7 may make up a fundamental part of innate cutaneous defense to fight off *S. aureus* infections and that a dysregulation of RNase 7 in general enhances the susceptibility toward *S. aureus* infections. However, this intriguing hypothesis has to be verified in further studies.

Chronic, recurrent, and persistent infections caused by *S. aureus* are often associated with the formation of the small-colony variant (SCV) phenotype. SCV have a longer generation time leading to small colonies on agar plates. It is believed that a switch from the wild-type phenotype to a SCV phenotype makes it possible for *S. aureus* to escape from host defense and to spread the infection (79). To proof the hypothesis that SCV are less susceptible to the cutaneous AMP-mediated killing activity, Gläser et al. used clinical *S. aureus* SCV strains and exposed them to either AMP directly or to stratum corneum extracts from healthy donors. In both setups the killing activity toward *S. aureus* isolates displaying the SCV phenotype was markedly reduced (43). These experiments demonstrated a less susceptibility of SCV to the antimicrobial activity of RNase 7 and other human skin-derived AMP. Thus, switching into the SCV phenotype may help *S. aureus* to subvert cutaneous innate defense through a higher resistance toward AMP such as RNase 7.

An increasing threat is the spread of MRSA. MRSA acquired resistance mechanisms against many antibiotics commonly used to treat *S. aureus* skin infections. In human skin equivalents infected with MRSA the expression of RNase 7 was induced, in particular in wounded skin (28). Of note, RNase 7 is able to dampen the growth of MRSA *in vitro* (12) and own unpublished results. These data indicate an important role of RNase 7 as cutaneous defense factor to control the growth of MRSA. It is an interesting hypothesis that patients with an impaired RNase 7-based cutaneous defense are more susceptible to the spread of virulent *S. aureus* including MRSA. Notably, the highly virulent strain USA300, a highly pathogenic MRSA causing severe skin and soft tissue infections, displays a comparatively low susceptibility toward the anti-staphylococcal activity of stratum corneum extracts (42). If a decreased activity of RNase 7 against USA300 contributes to this effect is speculative but not unlikely. This is supported by the fact that RNase 7 is a major component of the anti-staphylococcal activity of stratum corneum skin extracts (25).

As discussed above the presence of cationic amino acid residues endows RNase 7 with a positively charged N-terminal cluster that is required for antimicrobial activity. It is known that *S. aureus* has the capability to reduce its susceptibility toward the action of cationic AMP through the reduction of its negative surface charge. This can be achieved by the incorporation of D-alanine in its teichoic acids, a mechanism that lowered the susceptibility of *S. aureus* toward several skin-derived AMP including RNase 7 (42). It remains to be determined if the use of such strategies to lower the sensitivity toward the bactericidal activity of RNase 7 are associated with a higher pathogenic potential of distinct *S. aureus* strains.

### Wounds

An intact skin barrier is essential for the protection against potential pathogenic microorganisms. In case of wounding, a rapid and potent defense is pivotal. In this regard, it has been shown that RNase 7 is rapidly released on the skin surface after experimental superficial barrier disruption (23). In contrast, Dressel et al. detected no increase of RNase 7 expression in the margins of chronic wounds, whereas the expression of the AMP hBD-2 and psoriasin was strongly induced. This led to the hypothesis that an insufficient expression of RNase 7 in chronic wounds may contribute to disturbed wound healing (80). Zanger et al. reported on a 50% lower RNase 7 gene expression in skin 3 days after sterile wounding (81). This in turn supports the idea that RNase 7 acts primarily as a component of the early and primary cutaneous defense response after injury (81). Clearly, more studies are needed to decipher the antimicrobial and immunomodulatory functions of RNase 7 in wounded skin.

### Mycobacterial Infections

It has been reported that RNase 7 exhibits antimicrobial activity against *Mycobacterium vaccae* at low micromolar concentrations (40). Furthermore, infection of airway epithelial cells with *Mycobacterium tuberculosis* led to induction of RNase 7 expression and an intracellular association of RNase 7 with *Mycobacterium tuberculosis* (50). This may suggest a direct antimicrobial effect of RNase 7 on *Mycobacterium tuberculosis*, but this has to be confirmed in further studies. Nevertheless, these initial studies give rise to the hypothesis that RNase 7 may play a role in infections caused by mycobacteria. Thus, it remains to be shown whether RNase 7 may also be involved in cutaneous mycobacterial defense (82).

## Outlook

**Figure 2:**
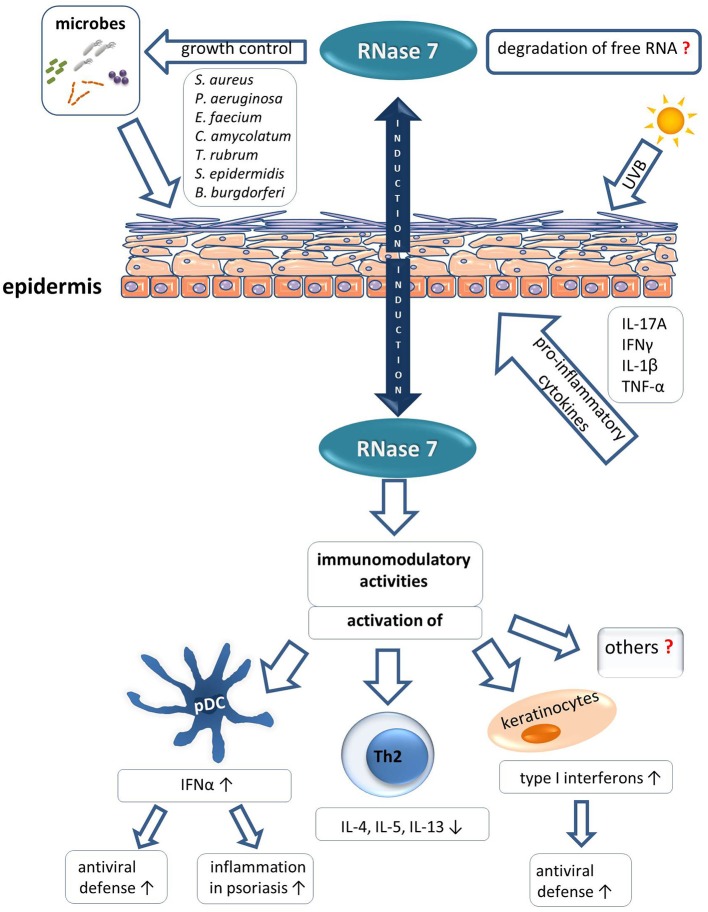
Role of RNase 7 in skin defense.

The role of RNase 7 in the skin is still emerging. However, as outlined in this review there is increasing evidence that RNase 7 plays an important role in innate cutaneous defense. This is mediated by the antimicrobial and immunomodulatory characteristics of RNase 7 (Figure 2 summarizes the role of RNase 7 in skin defense). It is likely that future studies will reveal novel immunomodulatory functions of RNase 7 and shed light on the as yet poorly understood importance of the enzymatic activity of RNase 7 in a physiological context. Moreover, it is of importance to further elucidate the potential link between specific infectious and inflammatory diseases and an impaired expression and/or function of RNase 7.

As mentioned in this review, functional studies as well as *in vivo* observations identified RNase 7 as a crucial factor to control the growth of the major cutaneous pathogen *S. aureus*. There is also evidence that other pathogens such as *P. aeruginosa* are affected by RNase 7. Thus, it is an interesting hypothesis that RNase 7 or optimized derivatives based on RNase 7 may have a high potential as anti-invectives. In this regard, the *in vitro* activity of RNase 7 against multi-resistant bacteria (12) may offer a promising alternative to fight off bacteria that are hard to kill by common antibiotics. Moreover, the targeted design of chimeric constructs combining selected parts of different molecules with high antimicrobial and ribonuclease activity, as recently reported for an RNase 3/1 hybrid construct (83), is a promising strategy. However, when considering RNase 7 for application as a therapeutic drug it is of great importance to evaluate the possibility that an excessive medical use of RNase 7 may induce the emergence of bacteria with an acquired resistance toward RNase 7. Such scenario could threaten our own innate host defense. In addition, the influence of immunomodulatory activities as well as the interaction of RNase 7 with the microbiota have to be considered when using RNase 7 in the treatment or prophylaxis of infections. In the case of atopic dermatitis, where *S. aureus* and elevated Th2 cytokines are major drivers of the disease, the capability of RNase 7 to kill *S. aureus* together with its influence on T cells to dampen secretion of Th2 cytokines may qualify RNase 7 as a beneficial drug to treat atopic dermatitis. Finally, the inducibility of RNase 7 gives rise to the speculation that a targeted induction of RNase 7 may offer a useful treatment or prophylactic option. Clearly, more studies are needed to unravel the physiological role of RNase 7 and to define its role in diseases and as a potential drug.

## Author Contributions

FR, SD, and JH wrote the original draft and edited versions. VK, RG, and TW edited versions. FR and JH prepared the figures.

### Conflict of Interest

The authors declare that the research was conducted in the absence of any commercial or financial relationships that could be construed as a potential conflict of interest.
